# Glucan–Resveratrol–Vitamin C Combination Offers Protection against Toxic Agents 

**DOI:** 10.3390/toxins4111301

**Published:** 2012-11-09

**Authors:** Vaclav Vetvicka, Jana Vetvickova

**Affiliations:** Department of Pathology, University of Louisville, 511 S. Floyd, MDR Bldg., Rm. 224, Louisville, KY 40292, USA; Email: jana.vetvickova@louisville.edu

**Keywords:** glucan, resveratrol, vitamin C, mercury, PFOS, PFOA

## Abstract

Biological immunomodulators are routinely evaluated as a natural source of molecules with profound effects on the immune system. They belong to a group of physiologically active compounds, collectively termed biological response modifiers. Most of the studies were focused on immune system stimulation. Recently, they have become the focus of studies seeking molecules that are able to overcome negative effects of various immunotoxins. This paper concentrates on the effects of a glucan/resveratrol/vitamin C combination on immunosuppressive effects of mercury and perfluorinated hydrocarbons. Effects described in this review have strong clinical potential, as environmental contaminants have adverse effects on all aspects of the immune system and represent a serious threat to the health of both humans and animals.

## 1. Introduction

Environmental contaminants having adverse effects on organs and tissues of the immune system represent a serious threat to the health of both humans and animals. It is well documented that such toxins increase host susceptibility to infections, compromise immunosurveillance and increase the chance of autoimmune diseases. These problems have led to efforts to identify sets of immunologically relevant end points and large epidemiologic studies [1]. 

Thimerosal has been used as a wound disinfectant and a preservative in vaccines for quite some time [2]. However, studies performed during the past several decades clearly established the immunosuppressive effects of various types of mercury [3,4]. Immunodepression was originally connected with primarily organic methyl mercury (for review see [5]). Since the scare of the possible effects of thimerosal in vaccines, mercury remains one of the most closely examined immunotoxins. Additional immunotoxins involve altrazine [6], bilirubin [7,8] or nickel [9]; however, the list of all potential immunotoxic agents is almost endless.

To identify the potentially immunotoxic compounds and to regulate their use and/or accumulation represents an important approach. Of similar importance is the need to find ways to overcome these negative effects on animal and human health. Moreover, with the ongoing steady rise of health care costs, the benefits of an inexpensive and natural compound would be enormous. With regard to mercury poisoning, most of the effort is traditionally oriented towards acute poisoning. Common treatments consist mostly of chelation therapy, which can be hazardous if administered incorrectly. In the case of perfluorinated carboxylates, the treatment consists of a series of steps that include gastric lavage, endoscopy and antidote. Again, much less attention is focused on low-dose poisoning. 

The purpose of this review is to provide new insight into our current knowledge of biological and immunological activities of a glucan–resveratrol–vitamin C combination and how these compounds might overcome the immunotixic effects of several known toxins. Thus far, no natural immunomodulator has been used in the treatment of low-dose effects of immunotoxins.

β1,3 glucans are structurally complex homopolymers of glucose, usually isolated from yeast and fungi. β1,3-glucan’s role as a biologically active immunomodulator has been well documented for over 45 years. Interest in the immunomodulatory properties of polysaccharides was initially raised after experiments indicated that a crude yeast cell preparation stimulated macrophages via activation of the complement system [10]. After extensive research, stimulative effects of glucans on cancer, infection, cholesterol level, and blood sugar levels have been demonstrated (for review see [11,12]). 

However, despite the clear and well-established biological effects of glucan, the search for even better results continues. Lately, more and more manufacturers have been experimenting with the preparation of various cocktails or mixtures of potentially bioactive powders. Recently, new studies appeared demonstrating that some molecules have synergistic effects when combined with glucan. Initial scientific studies have shown beneficial effects on both nonspecific and specific immunity when glucan was given in combination with vitamin C [13,14]. Additional work reported strong synergy between glucan and humic acid [15] and glucan and resveratrol [16]. Based on these data, we later focused on an evaluation of biological effects of a glucan–resveratrol–vitamin C combination and showed that this combination has superior immunological effects compared to individual components in cellular, humoral and anticancer immunity [17]. 

## 2. Effects on Mercury

For evaluations of the possible effects on mercury poisoning, we chose a treatment with a two-week daily dose of thimerosal corresponding to approximately 200 μg of mercury/kg. This treatment induced a systemic suppression of all tested reactions—from cellular (phagocytosis, NK cell activity, mitogen-induced proliferation and expression of CD markers) to humoral immunity (antibody formation and secretion of IL-6, IL-12 and IFN-γ) [18]. 

Originally, we tried to determine if glucan alone, as the immunologically most potent part of the combination, can change the direct toxicity of mercury. Studies of the simultaneous administration of mercury compounds and glucans began with changes in direct toxicity of mercury. In both cases, glucans significantly lowered the toxicity. As the direct toxic effects of mercury compounds are hypothesized to be caused by apoptosis, well-known inhibition of apoptosis caused by glucan might be the explanation. 

**Figure 1:**
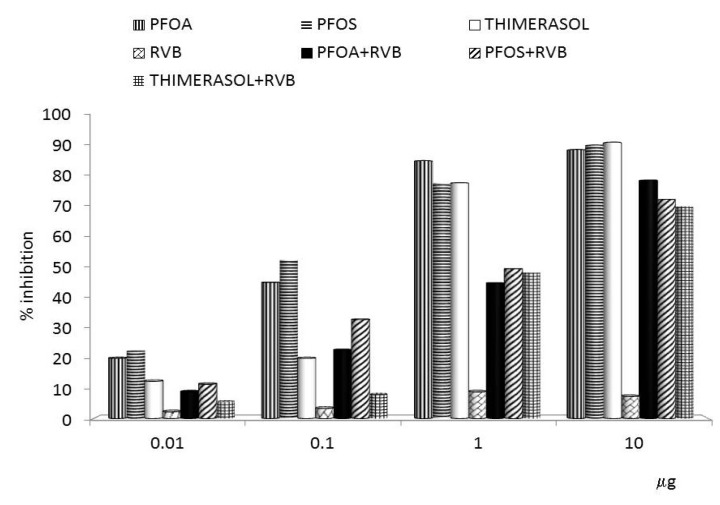
Effects of the glucan/resveratrol/vitamin C combination on proliferation of human monocyte cell line U937. PFOA—perfluoroctanic acid; PFOS—perfluooctane sulphonic acid; RVB—resveratrol/vitamin C/glucan combination.

In all our tests, glucan significantly lowered the toxicity of not only thimerosal, but also mercury acetate [18]. It is important to note that even if glucan managed to partially block the cytotoxic effects of mercury, it is possible that with long glucan application the level of immune reactions might be normalized. The broad range of affected reactions covering both branches of the defense reactions suggests strong and systemic restoration caused by glucan administration (Figure 1).

The exact mechanisms of this action are not known, despite extensive research on the immunological effects of glucan [11,19]. Since the direct toxic effects of mercury compounds were repeatedly suggested to be caused by apoptosis [3], inhibition of apoptosis caused by glucan [20] could be the explanation. In addition to direct stimulation of cells via Dectin-1 and CR3 (complement receptor 3) receptors (for review see [12,21]), glucans are known to alter some important genes and their transcription factors [22]. Furthermore, since mercury is known to cause inflammation and oxidative stress [23], intracellular mechanisms that involve antioxidant processes where glucan plays an important role [24] might be assumed as well [25]. 

**Figure 2:**
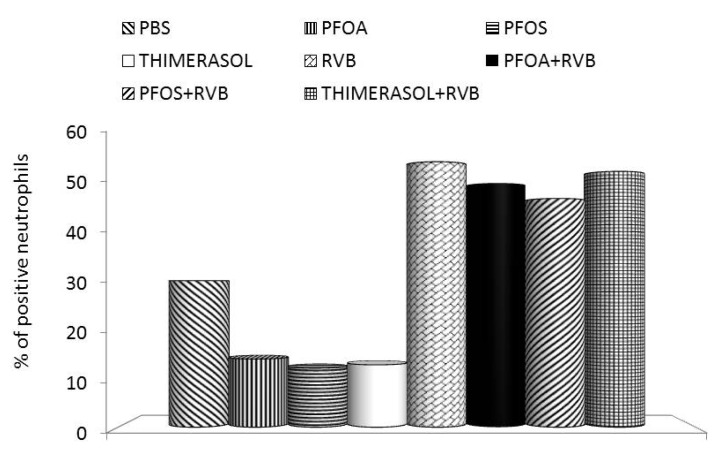
Effects of the glucan/resveratrol/vitamin C combination on phagocytosis of synthetic particles by mouse peripheral blood neutrophils. PFOA—perfluoroctanic acid; PFOS—perfluooctane sulphonic acid; RVB—resveratrol/vitamin C/glucan combination.

**Figure 3:**
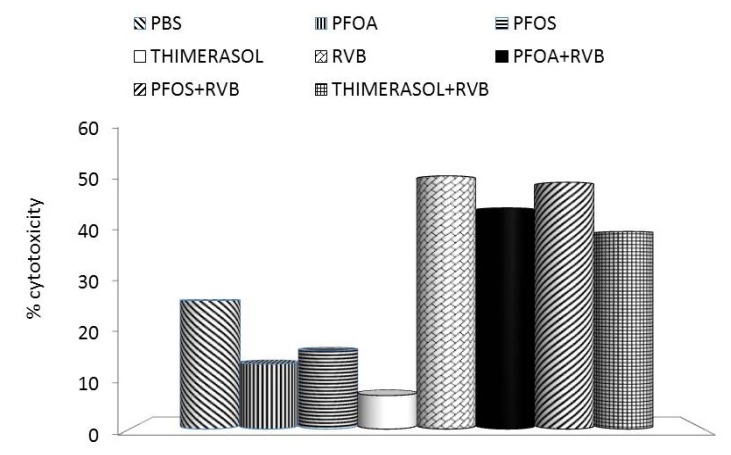
Effects of the glucan/resveratrol/vitamin C combination on NK cell activity of mouse splenocytes. PFOA—perfluoroctanic acid; PFOS—perfluooctane sulphonic acid; RVB—resveratrol/vitamin C/glucan combination.

**Figure 4:**
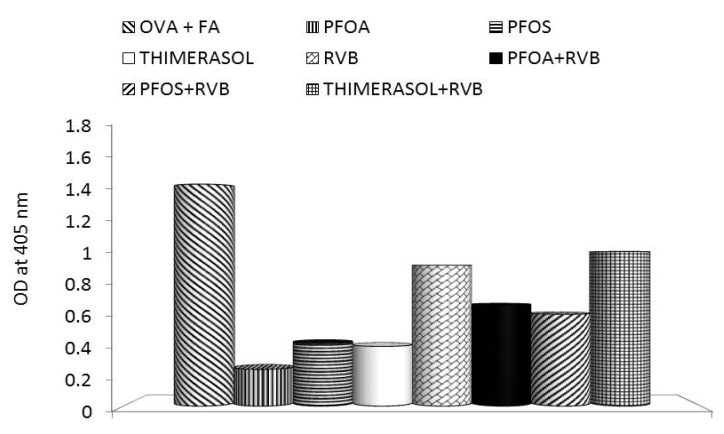
Effects of the glucan/resveratrol/vitamin C combination on *in vivo* antibody response to challenge with ovalbumin. PFOA—perfluoroctanic acid; PFOS—perfluooctane sulphonic acid; RVB—resveratrol/vitamin C/glucan combination.

Our results showed that thimerasol strongly inhibited the proliferation of human monocyte cell line U937. Simultaneous treatment with thimerasol and RVB 300 (resveratrol/vitamin C/glucan) decreased this suppression up to 50% in doses ranging from 0.01 to 1 μg/well (Figure 1). Thimerasol had direct effects on cell viability, but also on cellular immunity. When we tested the phagocytosis of synthetic microspheres by peripheral blood neutrophils, we found that thimerasol showed 58% inhibition of this function. When used simultaneously with RVB 300, only 3.7% inhibition was found (Figure 2). Similar results were found in NK (natural killer) cell activity (Figure 3). However, thimerasol extended its suppressive effects on humoral immunity as well. Figure 4 showed that thimerasol can also suppress humoral immunity and once again the addition of RVB 300 returned the antibody response to level of RVB 300.

## 3. Effects of PFCs

Perfluorinated hydrocarbons are currently used in thousands of different products. Two major classes of these compounds are perfluorinated carboxylates, such as perfluoroctanic acid (PFOA), and perfluoroctane sulfonates, such as perfluooctane sulphonic acid (PFOS). They have an extremely long lifetime in the environment and are detectable in the blood of both animals and humans [26]. Numerous studies showed that exposure to these compounds caused suppression of lymphocyte proliferative and NK cell activity [27], neutrophenia [28] and decreased production of some cytokines [29]. Readers seeking more information on immunosuppressive effects of these compounds are encouraged to read the comprehensive review [30]. Recently, Grandjean *et al.* reported reduced humoral response to immunization in children exposed to PFCs [31].

Polyfluoroalkyl chemicals have been used since the 1950s in numerous commercial applications and the exposure of the general U.S. population to PFCs is widespread. In this population, PFOS and PFOA serum concentrations were measurable in each demographic group studied [32]. The reductions in concentrations of PFOS and PFOA observed lately are most likely related to the discontinuation of industrial production by of some of the PFOS-related perfluorooctanesulfonyl fluoride compounds. Both PFOA and PFOS are frequently present in water-resistant textiles and sprays conferring water resistant properties. However, despite clear demonstration of the immunosuppressive effects of perfluorinated hydrocarbons, attention is focused entirely on methods to eliminate these compounds from their use. Our laboratory applied the data of glucan-derived protection *versus* mercury poisoning and tested the possible effects on immunosuppression caused by perfluorinated hydrocarbons.

Our studies showed that the strong suppression of proliferation of human monocyte cell line U937 caused by both PFOS and PFOA can be significantly reduced by simultaneous addition of RVB 300 (Figure 1). Similarly, phagocytic activity of blood neutrophils was significantly decreased, but the addition of RVB 300 returned the phagocytic activity to levels achieved with RVB 300 alone (Figure 2). Fifty percent NK activity reduction was fully restored by RVB 300 (Figure 3). In the final experiments, RVB 300 managed to partially overcome suppression of antibody formation caused by PFOS or PFOA exposure (65% and 71%, respectively, Figure 4).

## 4. Conclusions

Previous reports indicating that the addition of glucan resulted in significantly lower immunotoxic effects of mercury suggested that glucans can be successfully used as a natural remedy of low level exposure to immunotoxins [18]. Some of the glucan effects were profound even when used prophylactically [33]. It was later determined that simultaneous use of resveratrol and glucan showed stronger immunological effects on phagocytosis, faster restoration of spleen cellularity after experimentally induced leucopenia, and inhibition cancer growth *in vivo *[17,33]. Glucan use, in addition to humic acids, was verified by similar data. [15]. In addition, beneficial effects of glucan with vitamin C were also described [34]. Based on these results, we evaluated the immunological effects of a glucan/resveratrol/vitamin C combination and showed that this has the strongest impact of all commercially used natural compound combinations [35]. The mechanisms by which this combination improves the immunosuppressive effects of various toxins are yet to be determined. However, one possibility is the known protective effects on bone marrow, leading to increased production of immunocytes [36]. Another hypothesis suggested that as glucan directly increased several types of both humoral and cellular reactions, the glucan-stimulated cells would simply return the immune reactions to the previous levels. Data showing that in mice treated with the toxins and glucan/resveratrol/vitamin C, some reactions reached the same level as with the combination alone. These data, however, do not support this possibility.

All these data strongly suggest that natural immunomodulators such as glucan and some of glucan-based combinations represent an interesting and potentially clinically important way of blocking or at least partly restoring the immunosuppression of toxins such as mercury or perfluorinated hydrocarbons. From these observations, one can imagine that glucan-based modulators can be used for prophylactics in some cases of chronic immunopoisoning, strongly suggesting the need for further studies.
